# SEALNET: Facial recognition software for ecological studies of harbor seals

**DOI:** 10.1002/ece3.8851

**Published:** 2022-04-28

**Authors:** Zach Birenbaum, Hieu Do, Lauren Horstmyer, Hailey Orff, Krista Ingram, Ahmet Ay

**Affiliations:** ^1^ 3719 Department of Computer Science Colgate University Hamilton New York USA; ^2^ 3719 Department of Mathematics Colgate University Hamilton New York USA; ^3^ 3719 Department of Biology Colgate University Hamilton New York USA

## Abstract

Methods for long‐term monitoring of coastal species such as harbor seals (*Phoca vitulina*) are often costly, time‐consuming, and highly invasive, underscoring the need for improved techniques for data collection and analysis. Here, we propose the use of automated facial recognition technology for identification of individual seals and demonstrate its utility in ecological and population studies. We created a software package, SealNet, that automates photo identification of seals, using a graphical user interface (GUI) software to detect, align, and chip seal faces from photographs and a deep convolutional neural network (CNN) suitable for small datasets (e.g., 100 seals with five photos per seal) to classify individual seals. We piloted the SealNet technology with a population of harbor seals located within Casco Bay on the coast of Maine, USA. Across two years of sampling, 2019 and 2020, at seven haul‐out sites in Middle Bay, we obtained a dataset optimized for the development and testing of SealNet. We processed 1752 images representing 408 individual seals and achieved 88% Rank‐1 and 96% Rank‐5 accuracy in closed set seal identification. In identifying individual seals, SealNet software outperformed a similar face recognition method, PrimNet, developed for primates but retrained on seals. The ease and wealth of image data that can be processed using SealNet software contributes a vital tool for ecological and behavioral studies of marine mammals in the developing field of conservation technology.

## Graphical abstract

In this paper, we describe the successful application of our newly developed automated facial recognition software as a tool for ecological analysis of harbor seals (*Phoca vitulina*). We outline an emerging method of data collection and analysis that facilitates rapid interpretation of large photo datasets over wide temporal and geographic scales. In addition, we use this machine learning‐based technology in a preliminary ecological study in a wild population of seals in the Casco Bay region of Maine to demonstrate the effectiveness of this non‐invasive method for use in mark‐recapture and site fidelity studies in the field.

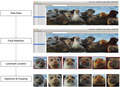

## 1 INTRODUCTION

The dynamic nature of marine ecosystems requires the monitoring of populations across a range of temporal and geographic scales to inform conservation efforts (Hindell et al., 2003). However, methods for long‐term monitoring of coastal species, including marine mammals, are often invasive, costly, and time‐consuming (Cunningham, 2009), underscoring the need for new techniques for systematic data collection and analysis. The automation of population survey tools can also improve the efficiency of long‐term monitoring by increasing reproducibility while decreasing cost and labor (Weinstein, 2018).

Due to the ecological and economical importance of marine mammals as predators (Aarts et al., 2019), systemic monitoring of these highly mobile species is critical for understanding their population dynamics across a large geographic range. Tagging methods to track marine mammals have been widely used in the past. However, these GPS‐monitoring devices are expensive, ranging from $1000 to $3000 for one device (*GPS and VHF Tracking Collars Used for Wildlife Monitoring*, 2017). In addition, the attachment of external devices may interfere with behaviors such as swimming speed, oxygen consumption, and metabolic rate, potentially corrupting the data collected or harming or disturbing the individual (Rosen et al., 2017). Aerial observation methods limit interference with marine mammal behavior, but this technique is also time consuming and expensive (Cunningham, 2009). Photo‐based identification techniques have been widely used in cetacean species and other marine mammals (Balmer et al., 2008; Cunningham, 2009; Elwen et al., 2009; Glennie et al., 2021; Rayment et al., 2009), and have the advantage of being non‐invasive, but manual interpretation of photographs is time‐intensive and often limited to small‐scale projects. In addition, diagnostic features may be difficult to photograph reliably in some species, like harbor seals, where pelage color and/or patterning changes over time and across seasons.

Harbor seals (*Phoca vitulina*) are important indicators of ecosystem health given their extensive overlap with human activities both in and out of the water, and these marine mammals are particularly vulnerable to increased anthropogenic activity (Allen et al., 1984). As top predators, seal populations affect ecosystem dynamics, with healthy populations in the Northern Atlantic likely decreasing competition among species such as flounder and dab (suborder *Pleuronectoidei*), and sole (family *Aciridae)*, and, in turn, influencing the balance of both ecologically and economically critical fish populations (Aarts et al., 2019). Increases in seal populations along the Atlantic coast of the United States have also increased the numbers of sharks that inhabit coastal waters, potentially affecting tourism revenue in addition to local ecosystems (O’Toole et al., 2020). Over the last century, the Atlantic coast populations of harbor seals in northeastern North America were heavily exploited. Following their protection under the Marine Mammal Protection Act of 1972, populations of harbor seals off the Northeast coast of the United States successfully rebounded to healthy population numbers. However, the steep decline in abundance prior to any legislation is evidence of the potential vulnerability of the population to acute or chronic ecological challenges.

As key regulators and indicators of ecosystem health (Heithaus et al., 2008), accurate monitoring of harbor seal populations and movement patterns is essential. Photographic identification of individual harbor seals will facilitate population measures, including measures of site fidelity and estimates of population size based on mark‐recapture methods. Harbor seals are relatively easy to monitor via photographic analysis as large numbers of seals can be observed non‐invasively as they congregate at “haul‐out” sites—areas where seals come out of the water to rest on rocky islets, allowing them to thermoregulate and avoid predation—which make them easily visible to researchers from afar (Honeywell & Maher, 2017). Some promising progress in photo ID techniques has been made using analysis of pelage markings, i.e., spots on the seal's coat that can be reliably used as diagnostic tools (Cunningham, 2009). However, the identification of individuals harbor seals based on pelage patterns is difficult due to the density of individuals at haul‐out sites and to changing coat patterns as seals mature or during annual molting. These difficulties highlight the need for a novel photographic identification technique that does not depend on whole‐body photography and that can be automated for the inexpensive, efficient classification of individuals.

Here, we propose the use of automated facial recognition technology as a system for the identification of marine mammals for ecological and population studies. We used deep learning methods and convolutional neural networks to develop SealNet, a redesign of the PrimNet software developed for primates. SealNet contributes the first marine mammal face recognition software to automate the process of seal identification for use by researchers in the field.

In this paper, we outline the creation of a graphical user interface (GUI), that allows the user to automatically select, align, and chip seal faces to facilitate the processing of raw data. Then, we develop a seal face recognition software to identify individual seals. We train and test this software on a wild population of Atlantic harbor seals in Casco Bay, Maine, U.S.A. We compare the performance of SealNet with its predecessor PrimNet and show that SealNet outperforms this software in the classification of harbor seals. SealNet provides a new, non‐invasive tool for tracking individual seals in ecological and behavioral studies.

## 2 METHODS

### 2.1 Photographic data collection

**FIGURE 1:**
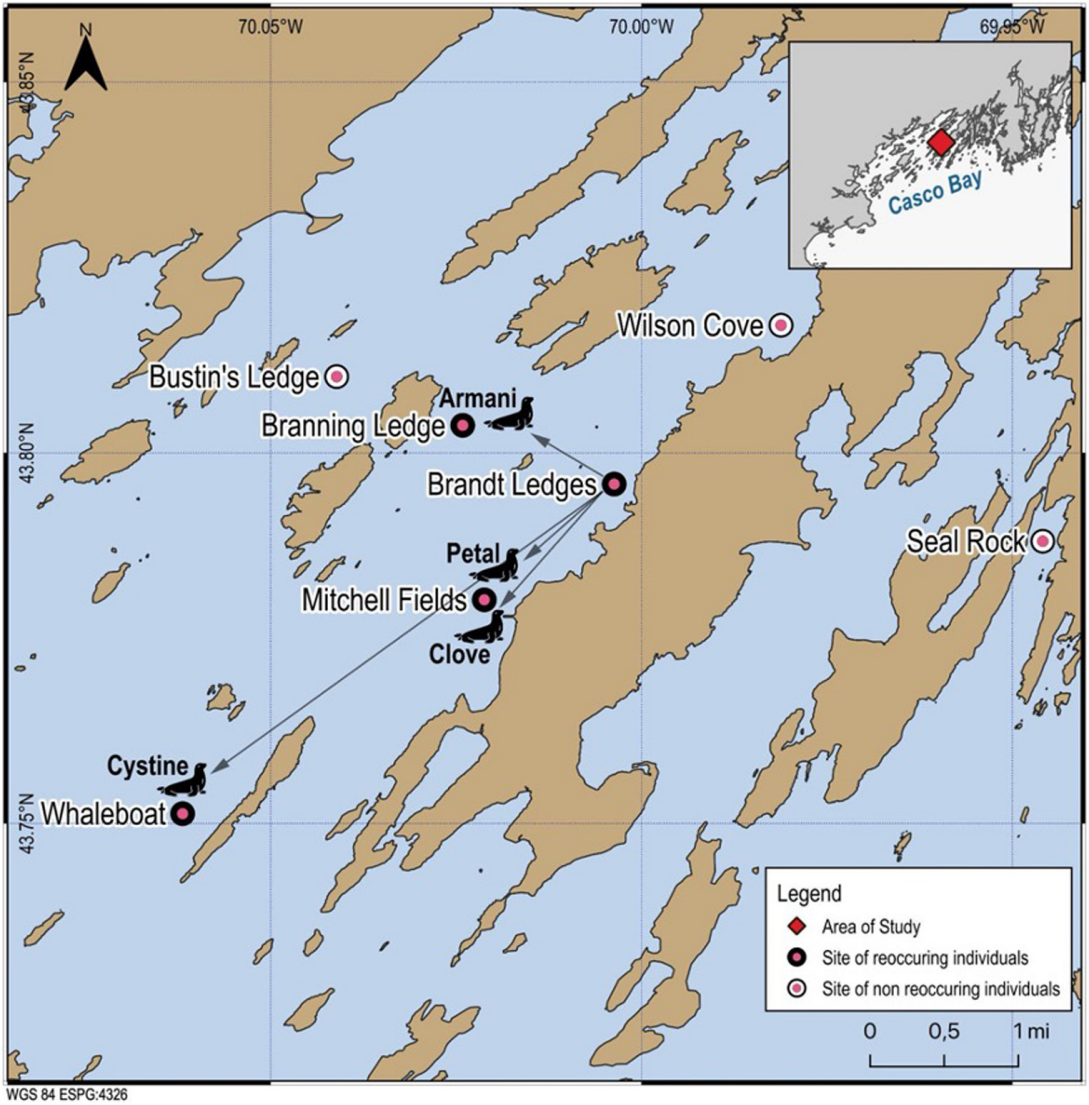
Harbor seal haul‐out sites photographed in preliminary study. In 2020, seal ‘015_Armani’ (photographed on Brandt ledges originally in 2019) was rephotographed on Branning Ledges; the seals ‘198_Petal’ and ‘211_Clove’ (both photographed on Brandt ledges in 2019) were rephotographed on Mitchell Field ledges; and the seal, ‘393_Cystine’, (photographed on Brandt ledges in 2019) was rephotographed on Whaleboat ledges

**FIGURE 2:**
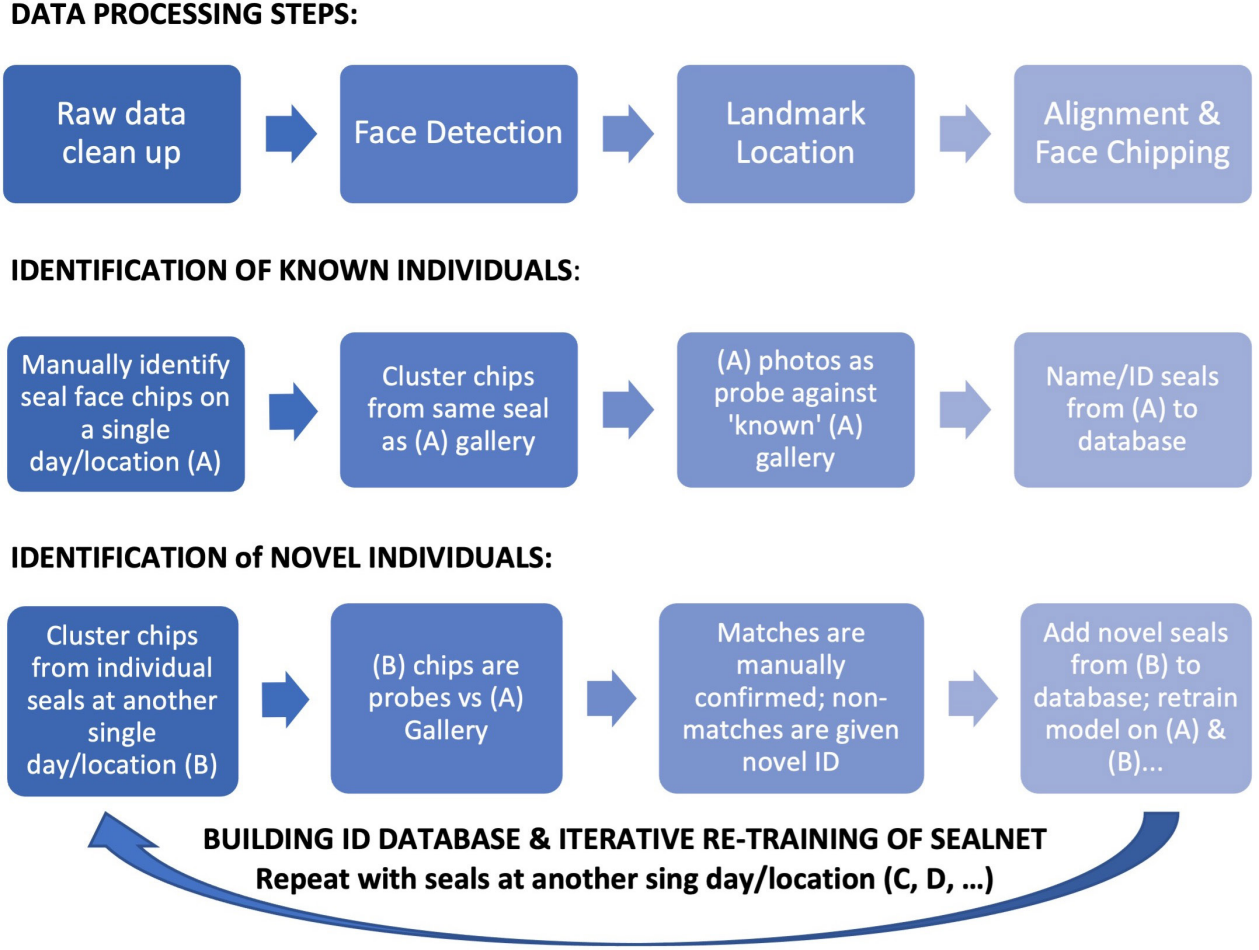
Summary of steps to create the final photo‐ID database using SEALNET

In the summers of 2019 and 2020, we captured 2267 photos across seven haul‐out sites around Casco Bay, Maine, U.S.A. (Seal Rock, Wilson Cove, Brandt Ledges, Mitchell Fields, Branning Ledge, Whaleboat, and Bustin's Ledge; see Figure 1). As we were optimizing the photographic data collection for developing and training SealNet, sites were visited only once, and there was no overlap in sites between 2019 and 2020 (Table 1). During a single visit to each site, we took photos for 30 min to one hour from a 22‐foot Eastern motorboat equipped with a 90‐horsepower engine, an open deck, and a low‐profile console. All site visits occurred in the summer (molting season) of each year, with exact dates dependent on weather and tides, as some of the sites are inaccessible at high tide. We used a Nikon COOLPIX P1000 digital camera with a 125x optical zoom. We photographed at a minimum distance of 54.9 m (60 yards) from haul‐out sites with the engine in low throttle or off to create minimal disturbance to the seals. We took multiple photographs of each individual seal as the boat drifted past the site. Below, we describe the steps of the pipeline for the processing of photographic data and the development of SealNet and the database of individual seal IDs; these steps are outlined in Figure 2.

**TABLE 1:** Dataset summary

Year	Date	Location	Total # Seals (# Chips)	Unique Seals (# IDs)
2019	7/16	Brandt Ledges	50	11
	7/20	Brandt Ledges	56	17
	7/24	Seal Rock	45	8
	7/27	Wilson Cove	19	4
	7/30	Bustin's Ledge	15	3
2020	7/01	Whaleboat	39	8
	7/10	Branning Ledges	820	197
	7/28	Whaleboat	33	8
	7/29	Branning Ledges	254	65
	7/31	Mitchell Fields	434	127

Note

The number of chips is the number of faces recognized by the facial recognition software. The number of IDs represents the number of individuals identified after grouping the chips according to individual.

### 2.2 Processing of photographic data

#### 2.2.1 Raw data cleaning

**FIGURE 3:**
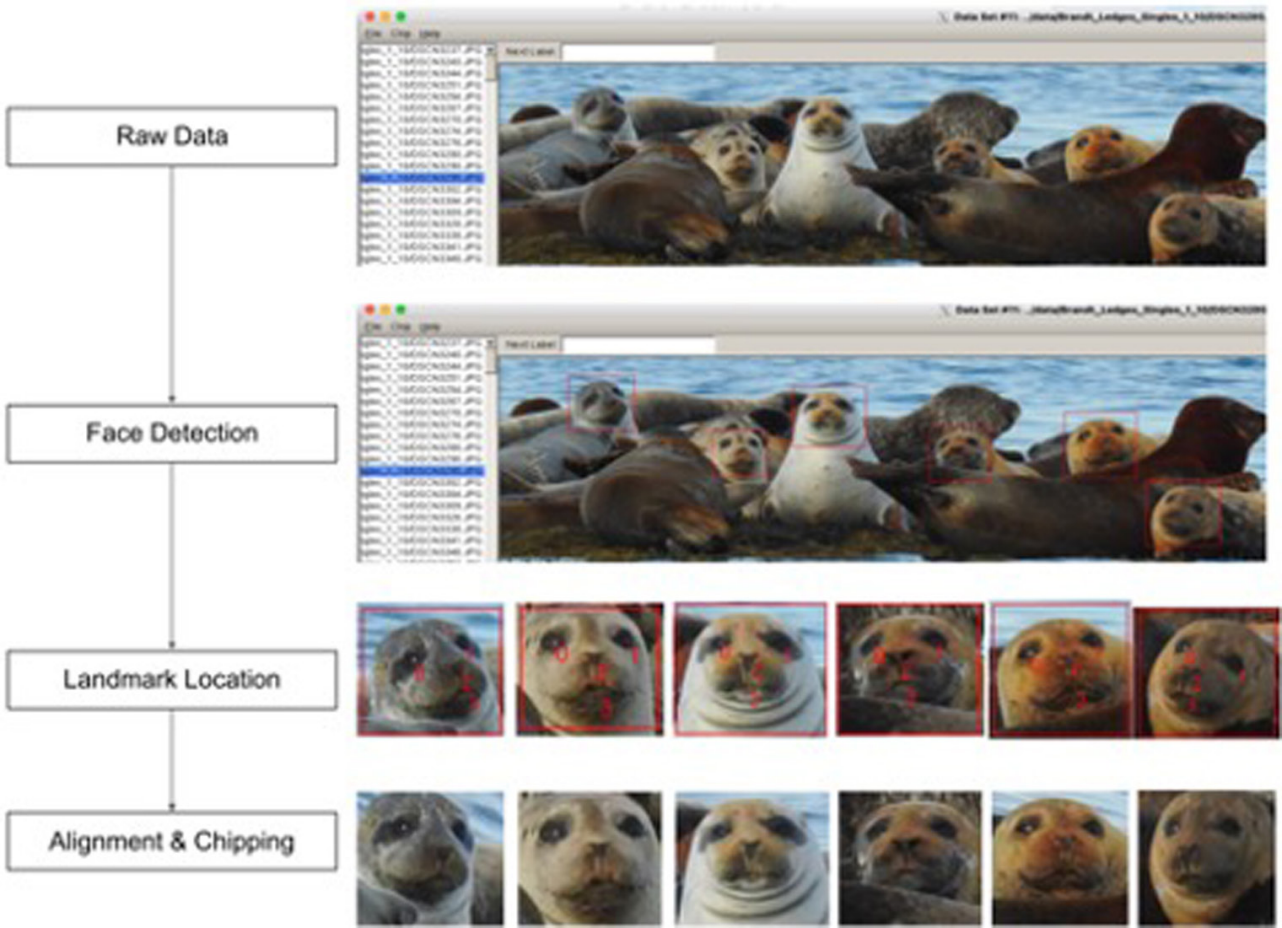
Summary of steps involved in face chipping. Step 1: Remove blurry and duplicate photos to create the raw photo dataset. Step 2: Run the automatic face detector to locate faces. Step 3: Manually locate the eye centers, nose, and mouth. Step 4: The GUI automatically aligns and chips all faces, saving output jpegs to a new folder. Step 5: Manually categorize chipped photos of the same seals into individual folders to be used for SealNet training

We manually processed the total number of photos in the database (>5000 images) to remove blurry photos, shots of sky or water, for a total of 2267 raw images. We then removed photo duplicates—images that were very similar to each other. We cropped each photo in the condensed dataset (*n* = 1752) to focus on the seal faces to minimize the amount of time the software takes to select faces. Once photos have been cropped, they are ready to be viewed in the graphical user interface and analyzed with the face detection software (steps outlined in Figure 3). For this preliminary study, we processed images across four haul‐out sites in 2019 and across three additional haul‐out sites in 2020 for a total of seven locations in Casco Bay (Table 1).

#### 2.2.2 Face detection

We created the graphical user interface (GUI) in C++ by modifying *imglab* tools for image annotation (King, 2009). We trained the interface to detect seal faces, allowing for automated detection of all seal faces in each photo. In addition, the GUI allows for the option to manually select seal faces by drawing boxes around valid faces in the application. A valid seal face is determined based on the quality and clarity of the image, as well as the angle of the complete seal face to the camera. Invalid faces are those that are too blurry, not facing the camera, or are partially obstructed; these can be marked by the user and will be ignored by the software. Variations in illuminations, lighting, and other conditions can introduce noise to the data and impede analysis. We next converted the photos to grayscale to help the model learn based on physical features of the face, which also serves to reduce overfitting during training. After all photos were aligned and chipped, we manually grouped photos of the same seals into folders by individual. To train our face detector, we selected 516 photos (10–20 seals faces per photo) from all locations in the 2020 dataset.

Our imglab based face detection software is a CNN network which uses Max‐Margin Object Detection (King, 2015) loss function. The first three layers of the network downsample the input images by 8 and output a feature map of 32 channels. This feature map will go through 4 more convolutional layers with batch normalization and Rectified Linear Unit (ReLU) as nonlinearity. The final output will only have 1 channel; a large value will indicate that the network has found an object at that location and vice versa.

**FIGURE 4:**
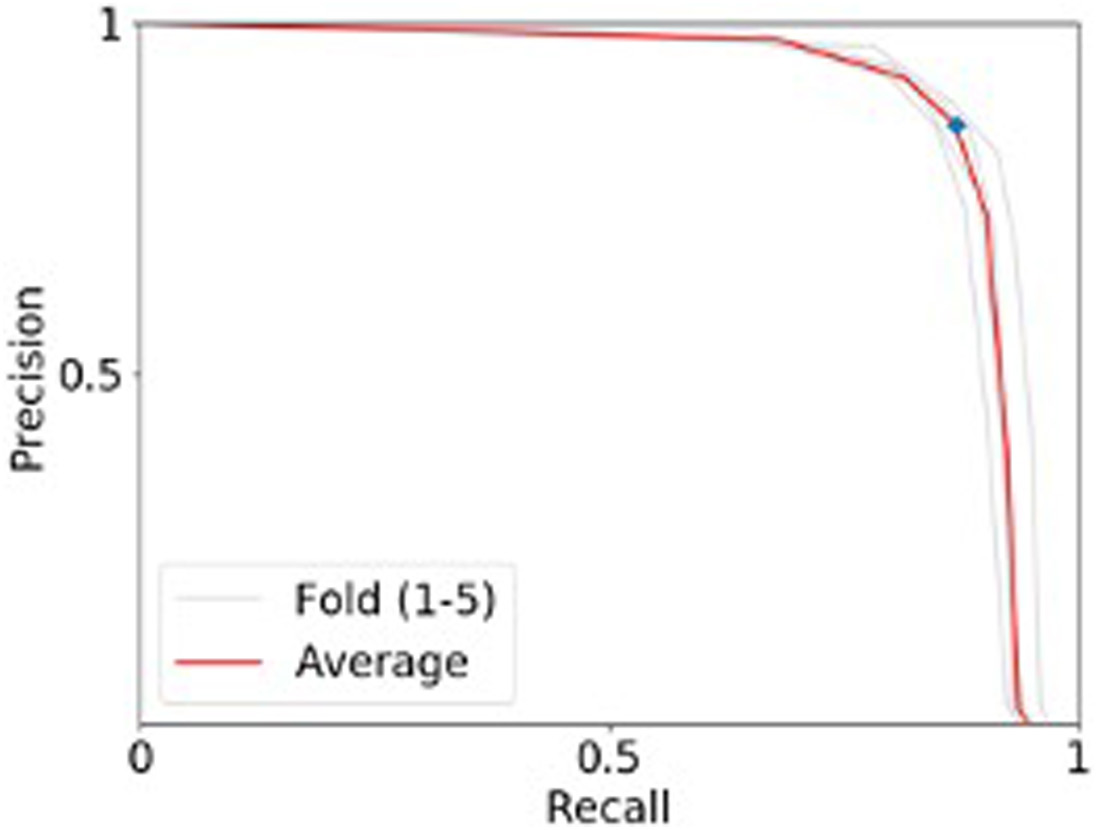
Precision‐recall curve of the seal face detector. The figure above shows the precision and recall at various thresholds of acceptance of the face detector. The precision‐recall was calculated from running 5‐fold cross‐validation on our dataset

Using the full 2020 dataset, we measured the accuracy of the model using 5‐fold stratified cross‐validation. Each strata (i.e., a single location and date) was split into 5 sections. For each fold, 4 of the 5 sections of each strata were combined as a training set while the remaining section of each strata were combined and used as a validation set. For each fold, the training set contained ~413 photos from all 5 locations, and the validation set contained ~103 photos from the same 5 locations. The accuracy of the face detector is measured by two metrics: precision (the percentage of predictions that are seal face) and recall (the percentage of total seal faces that are correctly predicted; Figure 4).

#### 2.2.3 Landmark location

Face alignment is critical for the accuracy of our face recognition software. As a result, prior to chipping the individual seal faces, we aligned them using the manually tagged eye (landmark) locations in each photo by performing in‐plane rotation to align the eyes along the x‐axis. Once the eyes are manually located in each photo, the GUI automatically aligns and chips the faces to the desired size (e.g., 112 × 112 pixels). We followed an approach similar to that used by the developers of LemurFaceID (Crouse et al., 2017) to align faces: Given $\left(l_{x},l_{y}\right)$ and $\left(r_{x},r_{y}\right)$ to be the center of the left and right eyes respectively, one can calculate the rotation matrix *M* to be used in an affine transformation of the image. Let $x=\frac{l_{x}+r_{x}}{2}$ and $y=\frac{l_{y}+r_{y}}{2}$ and $\theta =atan\left(\frac{r_{y}‐l_{y}}{r_{x}‐l_{x}}\right)$, so $\left(x,y\right)$ will be the location of the midpoint between the centers of the two eyes and θ be the rotational angle. Then *M* will be calculated as:

$$M=\left[\begin{array}{ccc} \cos\theta & ‐\sin\theta & x(1‐\cos\theta )+y\sin\theta \\ \sin\theta & \cos\theta & y(1‐\cos\theta )‐x\sin\theta \end{array}\right]$$

#### 2.2.4 Face alignment and chipping

Inter‐pupil distance (IPD) is the distance between the center of the two eyes, or $IPD=\sqrt{{\left(r_{x}‐l_{x}\right)}^{2}+{\left(r_{y}‐l_{y}\right)}^{2}}.$ We scaled each image automatically so that each eye would be 0.5×IPD away from the closest side edge and 0.6×IPD away from the top edge of the cropped face image. We chose these values by sampling 30 seal images and determining the optimum face to background ratio for facial recognition.

Thus, at the end of this step, each face image was rotated and resized to 112 × 112 pixels in preparation for facial recognition. The image label will contain information about its original image and the location within the original image from which it was chipped. Chips from multiple photographs of the same seal are clustered manually as a set that can act as probe images (if they are unknown) or gallery images (once they have been labeled with a name and ID number).

### 2.3 Development of SealNet

#### 2.3.1 SealNet architecture

**FIGURE 5:**
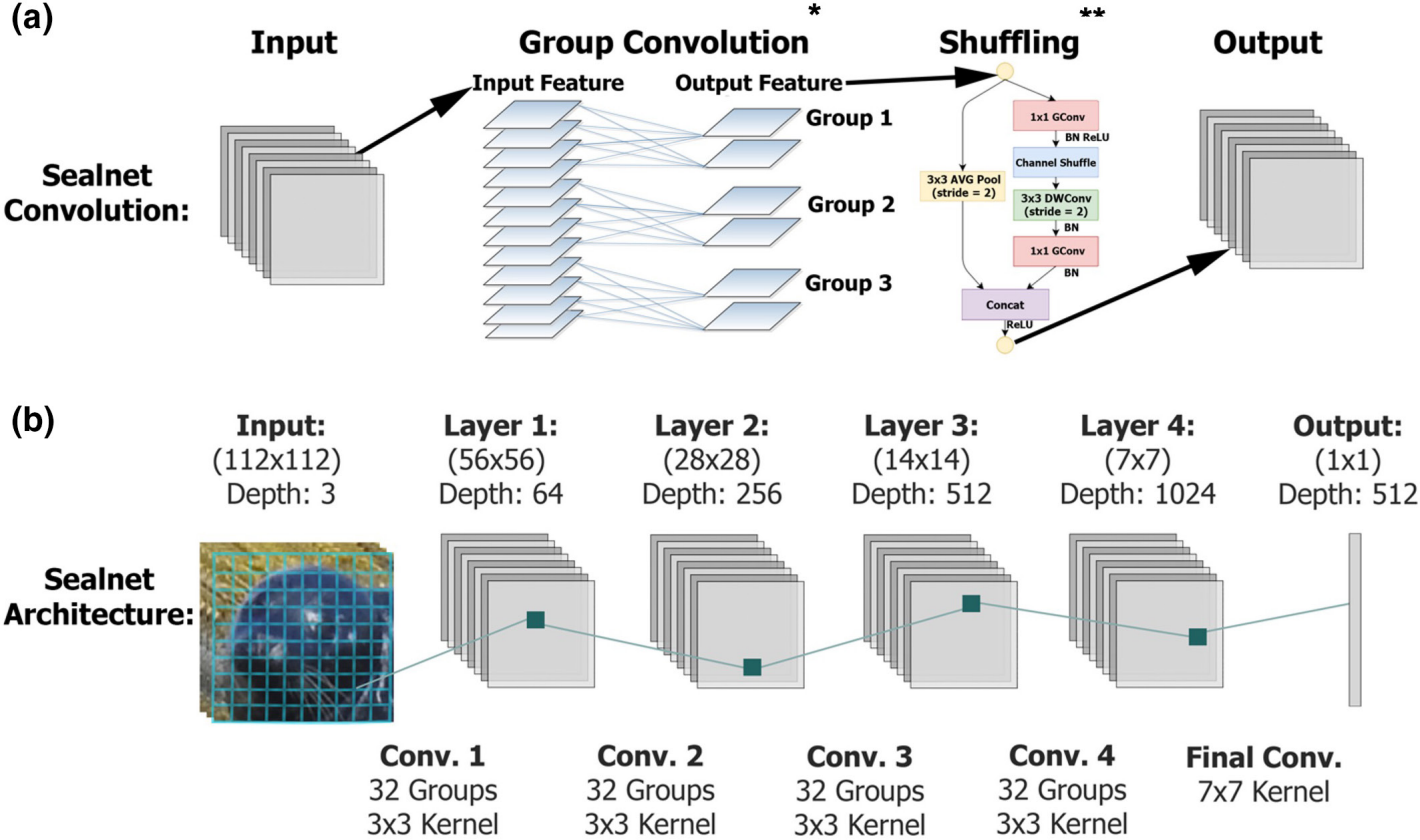
The architecture of SealNet’s recognition convolutional neural network. (a) The full operation of a single convolutional layer. Group Convolution is performed on the layer input. Shuffle operations are then performed on the Group Convolution’s output. Then, the output of this becomes the input to the next layer. (b) The architecture of SealNet’s layers and their respective parameters are pictured in the lower part of the illustration. The input is a photo of a seal and the output is a vector of length 512 representing the features extracted. *Image credit: Xu et al. (2020). **Image credit: Zhang et al. (2018)

The CNN‐based face recognition classifier is the main component of our software package. We train this classifier with photos that have been aligned, chipped, and normalized. Each input image underwent four convolutional blocks and a final bottleneck layer to output an embedded vector of length 512 that contained learned features of the input image (Figure 5). See Appendix S1 for additional details on the methodology involved in the development of SealNet.

#### 2.3.2 Validation of SealNet

In this biometric system, the probe set refers to the collection of biometric identities to be recognized, while the gallery set refers to identities that have been previously enrolled into the system. The gallery set acts as a database from which each probe identity will be searched. We measured the accuracy of SealNet with two standard recognition tasks: closed‐set and open‐set identification. In closed‐set identification, it is guaranteed that the identity in the probe is present in the gallery; whereas in open‐set identification, it is uncertain whether that is the case. Both closed‐set and open‐set refer to 1:N matching scenarios where each identity in the probe set will be searched against multiple identities in the gallery. The SealNet face recognition software produces a similarity score for each probe‐gallery pair and the result will be sorted in descending order so that the identity with the highest score will be the most likely matched candidate. We trained the model on an average of 485 chips of the same resolution for each fold of the closed‐set and on 533 chips with dimensions (112, 112) for the open‐set. We validated SealNet's face recognition capabilities using 5‐fold cross‐validation, with seals that have more photos than the number of folds.

To see how well our software performed compared to a previously developed facial recognition software, PrimNet, we trained and tested it and SealNet models using the same data and parameters We also measured how SealNet performs as we increase the size of our seal database (gallery set), for instance, by adding seals from new locations or new dates. Each time new data were added, we evaluated the model's closed‐set accuracy by running 5‐fold cross‐validation and calculating its average true identification rate.

#### 2.3.3 Developing the database of known individuals

Using a single folder of manually clustered chips from one site/location, we were able to create a gallery (A) of known individuals as each seal chip cluster was guaranteed to be a separate individual. We probed the (A) gallery with (A) images using the RecognitionGUI software, allowing for predictions of individuals. The RecognitionGUI software provides scores for each individual in the gallery, assigning them a number based on their facial biometric similarities to the individual in the probe. Similarity scores are provided for the top five ranked matches; top matches are confirmed by visual check in the output graphic. We added each known match to the database with a novel name and ID number. This (A) gallery was the foundation of our harbor seal ID database.

#### 2.3.4 Probing the database with new individuals

We subsequently processed folders of clustered chips (individual seals) from additional sites or days (B, C, D…) as probes to our original (A) gallery, one at a time. To add new individuals to the gallery database, a “match” probe photo (a photo from a new site or day that is already in the ID database) can be automatically merged to its matching file name and ID number. If the new individual does not match previous individuals in the database, a novel ID is created, and the seal photos and ID are added to the existing database. To speed up this process, we created a quick Python program, Tkinter GUI (Lundh, 1999) and added this program to the SealNet package.

## 3 RESULTS

### 3.1 Automatic face detection

We found that SealNet's face detector has a precision value (the percentage of predictions that are seal face) of 85.43% and a recall value (the percentage of total seal faces that are correctly predicted) of 86.94% after being trained on a dataset of 516 photos from one haul‐out site on a single day that contained 1178 valid seal faces. Figure 4 shows the accuracy of our model across different classification threshold levels for detecting a seal face. As the value of threshold decreases, the precision decreases to 0 while the recall approaches to 1. On the other hand, if threshold increases, the precision increases to 1 but the recall will decrease to 0. We chose threshold 0 for our face detector because it gives the best precision‐recall trade‐off.

We detected 49 false positives, that is, faces detected by SealNet that were not faces. Most were caused by vegetation or other parts of the seal that had face‐like shapes (Figure S1). SealNet missed on average 43 faces, mostly ones that were angled away from the camera (false negatives, Figure S2). We detected a total 408 unique seals, with an average of 2.9 photos per seal. Among these, 74 seals appeared in at least 5 photos.

### 3.2 Accuracy in seal identification

Our closed set data contained the 74 seals (same day/same location) that had at least 5 photos (607 photos in total). For each fold, the testing set contains one‐fifth of the number of photos of each of the 74 seals, and the training set contains the remaining photos of those “known” seals. We trained and tested both PrimNet and SealNet on the same data for each fold. Our average rank‐1 accuracy was 88(±0.03)% and our average rank‐5 accuracy was 96(±0.01)% across the 5‐folds (Table 2). PrimNet yielded 70% rank‐1 accuracy and 91% rank‐5 accuracy on the same dataset.

**TABLE 2:** Comparison of open‐set performance between SealNet and PrimNet for key metrics of model evaluation

F‐SCORE	Rank		TPR	FPR	FNR	TNR	Baseline	Accuracy	Precision	F‐Score
SealNet	R1	MEAN	0.678	0.032	0.322	0.968	0.892	0.945	0.656	0.663
		SD	0.019	0.012	0.019	0.012	0.011	0.011	0.094	0.048
	R5	MEAN	0.705	0.032	0.295	0.968	0.892	0.947	0.664	0.681
		SD	0.023	0.012	0.023	0.012	0.011	0.011	0.093	0.049
PrimNet	R1	MEAN	0.251	0.057	0.749	0.943	0.868	0.888	0.285	0.259
		SD	0.060	0.019	0.060	0.019	0.017	0.014	0.059	0.038
	R5	MEAN	0.281	0.057	0.719	0.943	0.868	0.891	0.307	0.285
		SD	0.063	0.019	0.063	0.019	0.017	0.014	0.047	0.036
Difference	R1	MEAN	0.427	−0.025	−0.427	0.025	0.023	0.057	0.371	0.405
	R5	MEAN	0.424	−0.025	−0.424	0.025	0.023	0.057	0.357	0.396

Note

In open‐set evaluation, any probe with a similarity score for its best match in the gallery less than the value of the threshold was rejected as an “imposter”. True Positives scored above the threshold and correct match was predicted within top “Rank” similarity scores (TPR). False Positives scored above the threshold but had no true match in gallery (FPR). False Negatives contained a match in gallery but had a top similarity score below the threshold, or the correct prediction for gallery member was not within the top “Rank” similarity scores (FNR). True Negatives had no match in the gallery and top predicted match had a similarity score below the threshold (TNR). Baseline accuracy is the accuracy score of the model assuming all probes were rejected. F1‐Score provides a better measure of propensity for incorrect classifications than accuracy, suited to unbalanced datasets.

Our open set data also included 74 seals with at least 5 photos and 571 photos from seals with fewer than 5 photos. Both PrimNet and SealNet models were trained and tested utilizing the same splits of data and equivalent parameters for number of epochs and batches per epoch to ensure fairness. F1 scores (defined as the harmonic mean of precision and recall), a measure of model performance for unbalanced datasets, showed a similar result with SealNet performing 39.6%–40.5% better than PrimNet (Table 2 and Table S1).

### 3.3 SealNet's performance on a growing dataset

We expanded the size of our closed set data five times, adding a new folder of seals and retraining the software each time, so our database increased from 194 to 406 unique seals. We calculated the average accuracy of both rank‐1 and rank‐5 training runs as shown in Table 3. Our data suggest that our model performs consistently (at the same accuracy level) as the size of our dataset increases.

**TABLE 3:** Iterative training accuracy for closed‐set data

# Seals	Rank 1 Accuracy	Rank 5 Accuracy
197	0.850	0.950
324	0.871	0.966
389	0.865	0.968
397	0.856	0.956
405	0.871	0.958

Note

The average rank‐1 and rank‐5 accuracy levels for each iterative training run following the probing of individuals from each date during 2020; accuracies are relatively robust to numbers of individuals.

**FIGURE 6:**
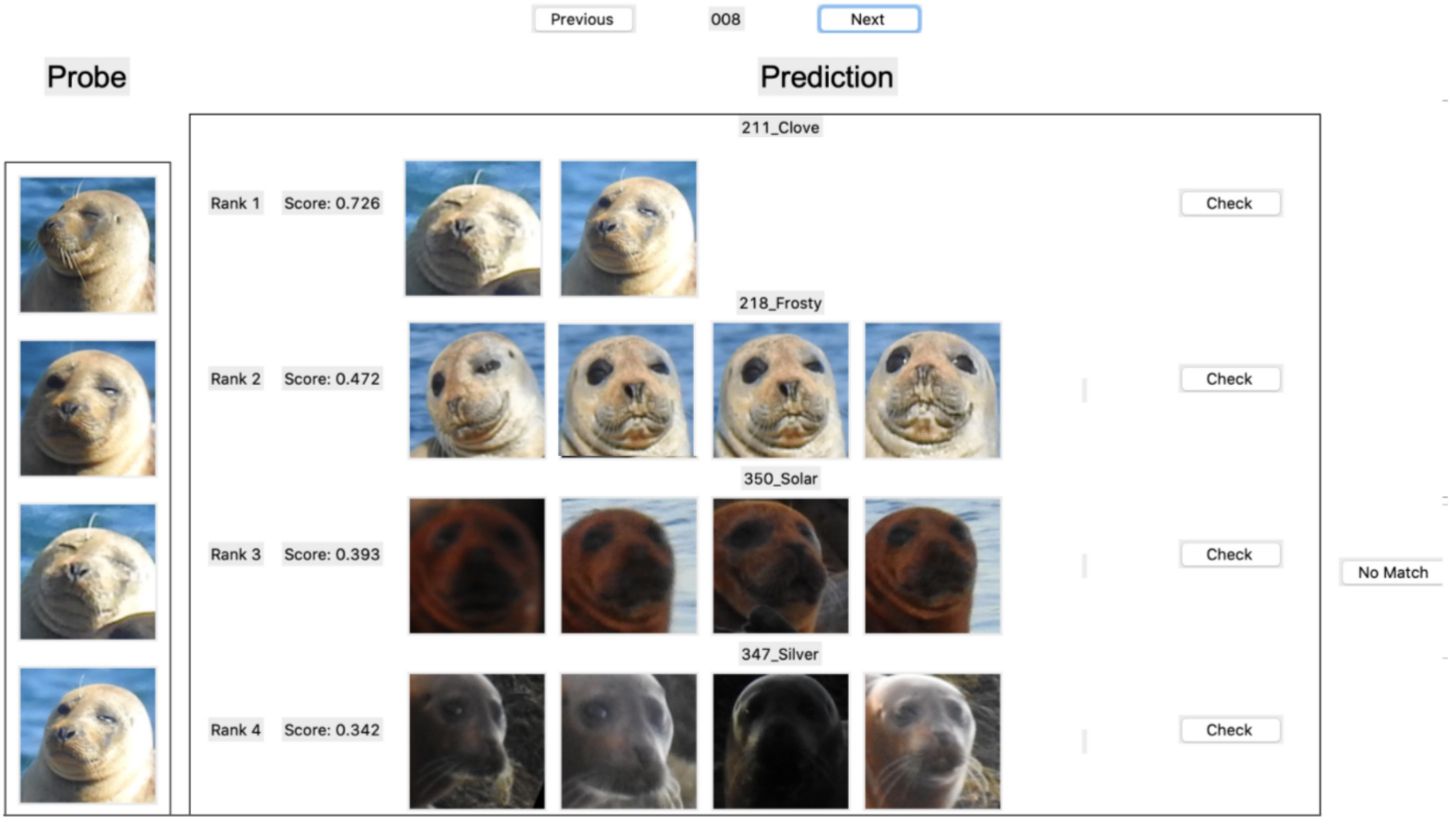
SealNet prediction of individual seals. The image above shows the probing process in which our recognition software matches individuals according to facial biometrics. Scores for each set of individuals in the training dataset are determined based on similarity in facial features to the probe individual, with the highest score indicating the most likely individual to be a match

Our Tkinter GUI allows for direct comparison of our existing seal database with new probed individuals, supported by similarity scores of the individuals in the gallery to the current probe (Figure 6). These similarity scores are based on facial biometrics of each individual, and they are determined after each image within a seal individual folder is compared to each image found in a gallery seal individual folder. On average, the similarity scores for individuals that are a match with the probe individual are approximately 0.65 with a SD of 0.1 (range 0.55–0.85). Similarity scores with individuals without a match range in similarity scores from 0.2 to 0.5.

### 3.4 Ecological results

SealNet identified four individual seals that were photographed in both 2019 and 2020: 015_Armani, 198_Petal, 211_Clove, and 393_Cystine. All four seals were originally photographed on Brandt Ledges in 2019 and were re‐photographed on Mitchell Fields (198_Petal and 211_Clove), Whaleboat (393_Cystine), or Branning Ledges (015_Armani) during the 2020 season. These preliminary findings suggest that some harbor seals exhibit site fidelity within local bays across years, and that there may be evidence of spatial connectivity among haul‐out sites.

## 4 DISCUSSION

Here we present the utility of a new software package, SealNet, an automated pipeline to non‐invasively identify individual seals in photographic images. We describe a novel face detector GUI trained to detect harbor seal faces and the development of a new neural network to classify individual seal faces. Following validation of the technique, we use SealNet in a preliminary study to explore site‐fidelity of harbor seals in the Casco Bay, Maine region of the northwestern Atlantic coast. Our initial validation analyses confirm the efficiency and accuracy of our facial recognition technology in the photo identification of an economically important coastal marine mammal.

### 4.1 Performance of automated SealNet pipeline

Our trained face detector had a precision of 85%, and a recall of 87% despite having very little restrictions on the position of the seals within the photo with the only limitation that both eyes of the seals are visible. This feature enables the successful use of SealNet software to conduct studies on animals in the wild without having the animals looking directly at the camera. The precision and recall of our detector could be increased by restricting the possible angle and pose of the seal, but this would limit the number of photos that meet such requirements in field studies.

For our recognition software, we have achieved high accuracy in both close‐set (rank‐1: 88% and rank‐5: 96%) and open‐set (rank‐1 and rank‐5: 93%) analyses, but there is still room for improvement. CNN‐based facial recognition software achieves identification accuracies of 93.8% with lemurs, 92.5% with chimpanzees (Schofield et al., 2019), and 97.27% with pandas (Chen et al., 2020). Another software, BearID, recently achieved close to 100% face chipping accuracy (number of faces detected in an unprocessed photo) despite an overall pipeline identification accuracy of 82.4% (Clapham et al., 2020). FaceNet (Schroff et al., 2015) which was trained on more than 3 million images of almost 10,000 unique human individuals, achieved an accuracy of almost 100%. Therefore, with a larger dataset with more photos per seal, it is possible that we can further improve our accuracy. Accuracy in studies utilizing pelage markings in seals is generally lower than facial recognition studies, with the rank‐1 accuracies of 59% and rank‐10 accuracies of 67% (Cunningham, 2009).

In a direct performance comparison of the classification task, SealNet performs better than PrimNet on average at all ranks with improved classification accuracy of up to 18% improvement at rank‐1 for closed‐set and 6% improvement for open‐set. It is also important to note that our model performs consistently well as our database increases in size. The consistent performance of our model demonstrates that SealNet generalizes well (i.e., overfitting is not an issue).

### 4.2 Preliminary ecological results

Using the SealNet facial recognition software package and a small, initial dataset sampled across two years (2019 and 2020) in Casco Bay, we identified four individuals in the datasets from both years, indicating a small degree of local site fidelity across years during the months of June and July. All four seals were found on the haul‐out site, Brandt Ledges, in 2019. In 2020, the four seals were photographed again within 1–3 nautical miles of Brandt Ledges: one was photographed on Branning Ledges, two were photographed at the Mitchell Field site, and one was photographed on the Whaleboat Island site. This result supports previous results suggesting site fidelity among harbor seals off the coast of NE Scotland (Cordes & Thompson, 2015). It is also interesting to note that two of the individuals found in the dataset from both years, Clove and Petal, were found together initially on Brandt Ledges on one day in 2019, and then found together again in 2020 at the Mitchell Field site. These results suggest that SealNet software may be useful in future long‐term studies of social relationships or aggregations in harbor seals. Previous studies have examined competitive relationships among harbor seals (Honeywell & Maher, 2017). However, further research is needed to examine other behavior‐related questions, including social fidelity, persistence of family groups, and other social dynamics.

Our preliminary ecological results suggest some site‐fidelity of harbor seals in Middle Bay as well as site‐fidelity to neighboring haul‐out sites within the bay. However, the initial photographic study was designed to provide the optimal photographic data for the development and training of SealNet. A more extensive ecological study is underway to determine the degree of site fidelity and spatial connectivity of haul‐out sites in this region. In addition, more extensive photographic data will help refine a population estimate for the number of seals utilizing Middle Bay. Current estimates of harbor seal abundance are outdated, suggesting a population of 38,014 individuals in the whole of Maine in 2001 (Gilbert et al., 2005), followed by an aerial survey done in 2012 determining a population of 75,834 individuals (Waring et al., 2015). Accurate local and regional population estimates are imperative to understanding the dynamics of seal abundance in relationship to anthropomorphic and climate changes to coastal marine environments, as well as the impact of an increasing great white shark population.

The use of facial recognition software to identify individuals in wild populations is a relatively new area of research and is primarily utilized in studies of land mammals such as lemurs and brown bears (Clapham et al., 2020; Crouse et al., 2017). Our research extends the use of such methods to marine mammal species. Facial biometrics are not the only measure that can be used for automated identification of seals. For example, a recent, groundbreaking study utilized pelage markings found on the seals coat to identify grey seal individuals near Wales (Langley et al., 2020). Given that coat patterns change across seasons during molting or over time in harbor seals, facial biometrics may offer an additional and/or more reliable method of identification. Thus, the development of facial recognition techniques for harbor seals allows for a rapid, non‐invasive means for detailed study of an economically and ecologically important species. Importantly, researchers can customize the software and the GUI to suit their own needs at each step of data collection—training the face detector for additional species, modifying the alignment procedure, or preprocessing images for face recognition.

### 4.3 Limitations

Although SealNet produced promising results, there are still limitations that need to be addressed. First, our SealNet software still requires some manual work during the data collection process—after running the automatic face detector, researchers are still required to manually locate the eyes, nose and mouth in order for the program to automatically align and chip the seal faces. Thus, one possible improvement that we can implement in the future is to add a landmark detector to be used in conjunction with the face detector. Secondly, to generate training data, researchers must manually group multiple face chips belonging to the same individual. Not only is this process laborious, it may be also error prone. A more sustainable approach would be to implement a classifier; however, researchers would still be required to manually check if the classification is accurate.

Although SealNet does well in closed‐set classification, open‐set verification performance could be improved by reducing the similarity scores between such seals. This success could be achieved with changes to our model architecture. However, the inherent complexity in any attempt to leverage specificity, while simultaneously avoiding overfitting, presents a difficult balance which all recognition models struggle to strike. Thus, the best approach to this problem would be to maximize the quantity and quality of information available to the model through preprocessing improvements prior to making changes to the CNN architecture itself.

## 5 CONCLUSION

We describe the development of SealNet, a novel facial recognition software package that includes an automated pipeline to detect individual seals from field photographs with high accuracy. The use of SealNet to identify individual harbor seals has multiple future applications to aid in decision‐making for conservation efforts, including assessments of seal abundance, evaluation of site fidelity within and across coastal regions, determination of trends in migration patterns, and the exploration of patterns in social behavior among harbor seals at haul‐out sites. The ease and wealth of data that can be collected with non‐invasive photography, coupled with the predictive ability of the SealNet to identify individuals, provides researchers with a robust toolkit that has the potential to transform ecological studies of wild populations of harbor seals. SealNet's ability to retrain and recognize additional marine mammal species provides a vital tool for ecological and behavioral studies of marine mammals in the developing field of conservation technology.

## CONFLICT OF INTEREST

The author(s) declare no competing interests.

## AUTHOR CONTRIBUTIONS

**Zach Birenbaum:** Data curation (equal); Investigation; Methodology; Software (equal); Validation (equal); Visualization (equal). **Hieu Do:** Data curation (equal); Investigation; Methodology; Software (equal); Validation (equal); Visualization (equal); Writing – original draft. **Lauren Horstmyer:** Investigation (equal); Methodology (equal); Visualization (equal); Writing – original draft (equal). **Hailey Orff:** Data curation (equal); Investigation. **Krista Ingram:** Conceptualization (lead); Investigation (equal); Methodology (equal); Project administration (lead); Resources (equal); Supervision (equal); Writing – original draft (equal); Writing – review & editing (lead). **Ahmet Ay:** Conceptualization (equal); Data curation (equal); Formal analysis (equal); Investigation; Methodology; Project administration (equal); Resources; Software (equal); Supervision; Validation (equal); Writing – original draft.

## Supporting information

Figure S1

Figure S2

Appendix S1

## Data Availability

*SealNet*is an open‐source application available on GitHub at https://github.com/zbirenbaum/SealFaceRecognition. The code and models are both also archived at Zenodo (Accession number: https://doi.org/10.5281/zenodo.6415595). Owing to file sizes, raw images will only be available upon request to the authors.
