# Supplementary material for: SEALNET: Facial recognition software for ecological studies of harbor seals
Source: Ecol Evol. 2022 Apr 28;12(5):e8851. doi: 10.1002/ece3.8851 (PMC9047973; doi:10.1002/ece3.8851)
Supplement: Supplementary file 1 — Figure S1 [file ECE3-12-e8851-s002.docx]

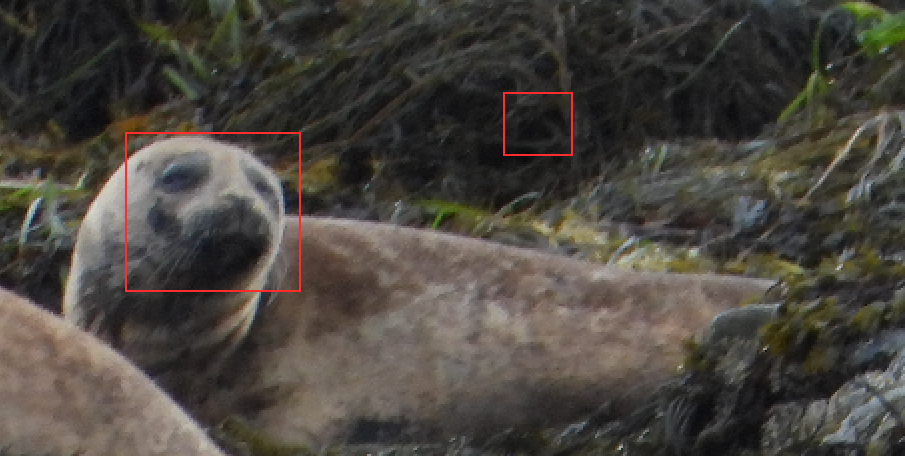


**Figure S1. False positive detection.** This figure includes a correctly identified seal face (left) versus an incorrectly identified non-face (right). Most false positive detections included similar vegetated areas that had a face-like shape.
