# Supplementary material for: SEALNET: Facial recognition software for ecological studies of harbor seals
Source: Ecol Evol. 2022 Apr 28;12(5):e8851. doi: 10.1002/ece3.8851 (PMC9047973; doi:10.1002/ece3.8851)
Supplement: Supplementary file 2 — Figure S2 [file ECE3-12-e8851-s001.docx]

**
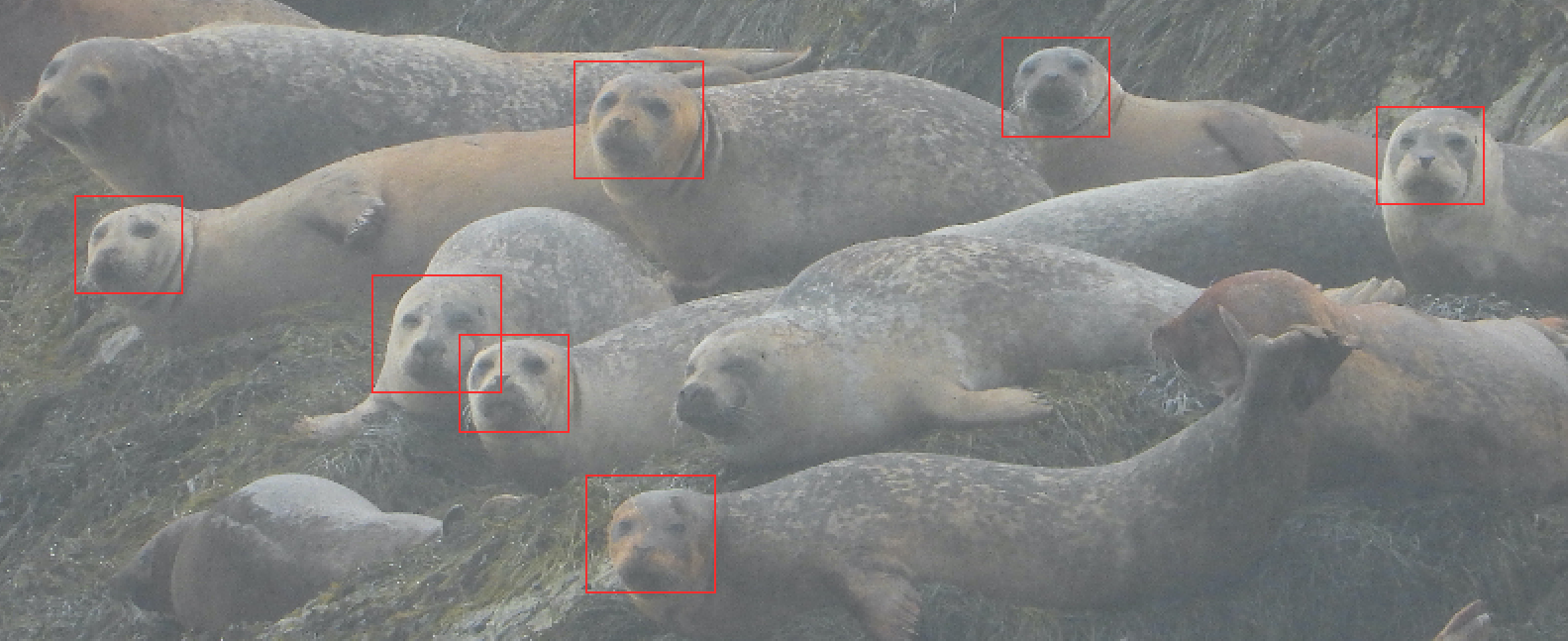
**

**Figure S2. False negative detection.** This figure includes missed detections, also known as false negatives. The face detector fails to detect a face where it is supposed to find one (top-left and center).
