# Supplementary material for: SEALNET: Facial recognition software for ecological studies of harbor seals
Source: Ecol Evol. 2022 Apr 28;12(5):e8851. doi: 10.1002/ece3.8851 (PMC9047973; doi:10.1002/ece3.8851)
Supplement: Supplementary file 3 — Appendix S1 [file ECE3-12-e8851-s003.docx]

**Supplementary Files**

**Supplementary Methods for SealNet Development**

**Automatic Face Detection**

Each detection has a confidence level associated with it. A higher confidence value indicates that it is more likely to be a valid seal face rather than a false alarm. Only detections that have a higher confidence value than our given threshold will be considered valid seal faces. Therefore, if the threshold is negative infinity, the precision decreases to 0 while the recall approaches to 1. On the other hand, if the threshold is positive infinity, the precision increases to 1 but the recall will decrease to 0. The precision-recall curve shows how these two values change as we change the threshold from negative infinity to positive infinity (Figure 4).

In our face detector software, we chose a threshold value of 0 because it gives the best precision-recall trade-off.

**SealNet Architecture and Training**

We trained the CNN for 100 epochs, using mini-batch gradient descent and a batch size of 16. We started with a learning rate of 0.01 and used ADADELTA optimizer (Zeiler, 2012) for our gradient descent. Each convolutional block contained a convolutional layer with a kernel size of 3x3 and a stride of 1 followed by a max-pooling layer with a kernel size of 3x3 and a stride of 2 (Figure 5). These blocks may contain an additional Squeeze and Excitation (SE) block that performs feature recalibration. The addition of SE blocks to our CNN helped the model better learn the interdependencies between channels, highlighting informative features while disregarding unimportant features (Hu et al., 2017). As proposed by PrimNet, our convolutional block also employs group convolutions followed by channel shuffling to make the network sparser to reduce vulnerability to overfitting (Deb et al., 2018). SealNet was trained using a GeForce RTX 2080 TI graphics card that took about 4 minutes and 33 seconds on average to finish training each fold.

**Validation of SealNet (with open- and closed-set identification)**

For closed-set identification, in each fold, we summarized the accuracy of our system using a Cumulative Match Characteristic (CMC) curve which plots the True Positive Identification Rate (TPIR) against the ranking of seals. TPIR measures the probability of observing a correct match within each rank. A correct match between probe *p* and an identity *g* in the gallery has rank *k* if the similarity score between *p* and *g* is the *k*th largest score (Stan Z. LiAnil & Jain, 2011).

For open-set identification, prior to splitting the dataset into 5 folds, we randomly select half of the seals with enough photos to be eligible for training, and put them, along with all seals lacking sufficient data to be included in training, into each of the testing sets as new seals. This method of training exclusions provided the best balance between the quantities of open-set testing photos and training photos. Whenever a probe-gallery pair’s similarity score exceeded our acceptance threshold, we “accepted” that individual; i.e., marked it as having been seen before (i.e the probe has a match in the gallery), while any probe with a similarity score that was less than the threshold value was rejected as an imposter.

Using the information on whether the probe was truly an imposter or not, all probes were categorized as follows: True Positives (TP) scored above the threshold and correct match was predicted within top “Rank” similarity scores. False Positives (FP) scored above the threshold but had no true match in gallery. False Negatives (FN) contained a match in gallery but had a top similarity score below the threshold, or the correct prediction for gallery member was not within the top “Rank” similarity scores. True Negatives (TN) had no match in the gallery and top predicted match had a similarity score below the threshold. Accuracy is measured as the ratio between the sum of TP and TN over all queries, which is equivalent to $\frac{TP+TN}{TP+TN+FP+FN}$. This formula is identical for open and closed set, but since the closed set inherently has no ‘True Negatives’ or ‘False Positives’, the closed set accuracy computation can be simplified to $\frac{TP}{TP+FN}$.

While it may appear strange that the open set scored higher accuracies than the closed set during evaluation, this can be explained intuitively by examining how the accuracy is defined for the open and closed set analyses. The probe has a match in a closed set, thus it is a potential true positive. As a result, the only way to improve accuracy in the closed set is to correctly identify more seals. Accuracy in an open set can increase when more seals are correctly identified or when more seals are correctly rejected.

Assume the model was given an open dataset containing 100 probes with a match in the gallery and 900 probes with no matches in the gallery. If the model accepts all seals (threshold of 0) and then classifies them, the accuracy would be at most 10%, assuming all seals were correctly classified. If the model rejects all seals and classifies none (threshold of 100), the accuracy is guaranteed to be 90%, because 900/1000 seals were correctly rejected. Because the threshold for each model was chosen for fairness purposes based on the F1 score it elicits, we recommend that this phenomenon be considered and readers focus on other metrics such as F1 score, True Positive Rates, and the difference in False Negative Rates, rather than accuracy, which is not a high quality metric of performance for open set evaluations for the reasons stated above.

**Performance Comparison of SealNet with PrimNet:**

To see how well our software performed compared to a previously developed facial recognition software, PrimNet, we trained and tested it and SealNet models using the same data and parameters. To further ensure fairness, for open set performance comparison, we tested the Rank-1 F1-Score results for each model at all threshold values in 0.01 increments and present the values for the run with the highest score. The Rank-5 scores presented use the same threshold as the best performing rank one, with loosened constraints for being classified as a True Positive. We used F1- Scores as there were only 74 seals with enough photos to meet the minimum requirements for being included in the gallery, while 571 photos were of seals with too few photos to have a corresponding gallery image. Because F1-Score provides a better measure of propensity for incorrect classifications than accuracy it is more applicable to imbalanced datasets like ours. Open set performance comparison between SealNet and PrimNet with thresholds determined by ~1% FAR instead of optimal F1-score are available below. Due to data limitations and the goal of our model, it may not be as representative of real-world performance as the F1-score table provided in the main text (Table 2). Baseline accuracy is the accuracy score of the model assuming all probes were rejected. TPR, or true positive rate, is the most intuitive measure of model performance, and shows the proportion of correctly classified probes at a given threshold.

**Supplementary Table 1.** Comparison of open-set performance between SealNet and PrimNet for key metrics of model evaluation. SealNet and PrimNet open-set performance for key model evaluation metrics are compared. During open-set evaluation, any probe with a similarity score for its best match in the gallery less than the threshold value was rejected as an 'imposter.' In this case, we used a threshold score that gives 1% False Accept Rates (FAR) for each run. True Positives scored above the threshold, and the correct match was predicted among the top "Rank" similarity scores (TPR). False Positives had a score above the threshold but no true match in the gallery (FPR). False Negatives had a match in the gallery, but a top similarity score lower than the threshold, or the correct prediction for a gallery member was not among the top "Rank" similarity scores (FNR). True Negatives had no matches in the gallery, and the top predicted match had a similarity score that was less than the threshold (TNR). The model's baseline accuracy is the accuracy score if all probes are rejected. F1-Score, which is better suited to unbalanced datasets, provides a better measure of propensity for incorrect classifications than accuracy.

| FAR | Rank |  | TPR | FPR | FNR | TNR | Baseline | Accuracy | Precision | F-Score |
| --- | --- | --- | --- | --- | --- | --- | --- | --- | --- | --- |
| SealNet | R1 | MEAN | 0.410 | 0.009 | 0.590 | 0.991 | 0.913 | 0.946 | 0.800 | 0.541 |
|  |  | SD | 0.046 | 0.000 | 0.046 | 0.000 | 0.000 | 0.004 | 0.018 | 0.045 |
|  | R5 | MEAN | 0.427 | 0.009 | 0.573 | 0.991 | 0.913 | 0.947 | 0.806 | 0.557 |
|  |  | SD | 0.050 | 0.000 | 0.050 | 0.000 | 0.000 | 0.004 | 0.018 | 0.046 |
| PrimNet | R1 | MEAN | 0.108 | 0.009 | 0.892 | 0.991 | 0.913 | 0.922 | 0.511 | 0.179 |
|  |  | SD | 0.020 | 0.000 | 0.020 | 0.000 | 0.000 | 0.002 | 0.048 | 0.031 |
|  | R5 | MEAN | 0.112 | 0.009 | 0.888 | 0.991 | 0.913 | 0.922 | 0.521 | 0.184 |
|  |  | SD | 0.017 | 0.000 | 0.017 | 0.000 | 0.000 | 0.001 | 0.040 | 0.026 |
| Difference | R1 | MEAN | 0.302 | 0.000 | -0.302 | 0.000 | 0.000 | 0.024 | 0.288 | 0.362 |
|  | R5 | MEAN | 0.315 | 0.000 | -0.315 | 0.000 | 0.000 | 0.025 | 0.285 | 0.373 |

**References:**

Deb, D., Wiper, S., Gong, S., Shi, Y., Tymoszek, C., Fletcher, A., & Jain, A. K. (2018, July 2). Face recognition: Primates in the wild. *2018 IEEE 9th International Conference on Biometrics Theory, Applications and Systems, BTAS 2018*. https://doi.org/10.1109/BTAS.2018.8698538

Hu, J., Shen, L., Albanie, S., Sun, G., & Wu, E. (2017). *Squeeze-and-Excitation Networks*. http://arxiv.org/abs/1709.01507

Li, S. Z. & Jain, A.K. (Eds.). (2011). *Handbook of Face Recognition* (2nd Edition). Springer.

Zeiler, M. D. (2012). *ADADELTA: An Adaptive Learning Rate Method*. http://arxiv.org/abs/1212.5701
